# Descriptive Characteristics of Multiple Myeloma Patients in King Abdulaziz Medical City National Guard

**DOI:** 10.7759/cureus.52692

**Published:** 2024-01-21

**Authors:** Sultan Alqahtani, Lama Alyabis, Hissah Alyabis, Nouf Al Qurashi, Rose Almadi, Majd Alsoman, Mohsen Alzahrani

**Affiliations:** 1 Department of Basic Medical Sciences, King Saud Bin Abdulaziz University for Health Sciences, College of Medicine, Riyadh, SAU; 2 Research, King Abdullah International Medical Research Center (KAIMRC), Riyadh, SAU; 3 College of Medicine, King Saud Bin Abdulaziz University for Health Sciences, Riyadh, SAU; 4 Department of Oncology, Division of Stem Cell Transplantation and Cellular Therapy, King Abdulaziz Medical City, Riyadh, SAU

**Keywords:** laboratory characteristics, clinical characteristics, mortality rate, prevalence, multiple myeloma

## Abstract

Background

Multiple myeloma (MM) is a hematological malignancy characterized by the production of monoclonal immunoglobulin. It is the second-most common hematological malignancy. The survival rate varies depending on age at diagnosis, comorbidities, and treatment.This study aims to assess the prevalence of clinical and laboratory characteristics among multiple myeloma patients.

Methods

This is an observational study of multiple myeloma patients who were admitted to King Abdulaziz Medical City - National Guard between January 2015 and December 2020. Patient records were reviewed to derive clinical and laboratory characteristics. Descriptive data analysis and survival analysis were obtained using SPSS.

Results

Our study included 151 patients, 95 of whom were males and 56 were females, and the mean age of diagnosis with MM was 62.6 (SD = 13.4). Among 151 MM patients, the most common clinical signs were bone lesions and renal disease, with a percentage of 66.9% and 46.4%, respectively. Death rates throughout the time of study conduction were 19.2%, accounting for 29 patients, and the median overall survival was 5.1 years with a 95% confidence level. Testing the association between survival rates and gender showed that death rates in females were significantly higher than in males (p-value = 0.023). Patients with anemia had a significantly higher hazard ratio in both unadjusted and adjusted analyses (aHR = 2.61; 95% CI = 1.21-5.65).

Conclusion

There was a relationship between survival and gender, which suggests a protective factor favoring the male gender. Clinical and laboratory characteristics, including bone marrow lesions, anemia, and renal disease, were the initial presentation; thus, a detailed history focused on symptoms should be taken when any of these symptoms are reported.

## Introduction

Multiple myeloma (MM) is a cancer of plasma cells, a type of immune cell found in the bone marrow [1]. Plasma cells produce antibodies, also known as immunoglobulins, to help the body fight infection and disease. In MM, the cancerous plasma cells grow out of control and produce an excess of monoclonal immunoglobulins, which are abnormal antibodies [2]. These antibodies do not function properly and can accumulate in the body, leading to a range of symptoms and complications. MM is typically diagnosed through a combination of laboratory tests and imaging studies. The presence of high levels of monoclonal immunoglobulins in the blood or urine, as well as abnormal plasma cells in the bone marrow, can be used to diagnose MM [3]. Other diagnostic tests may include imaging studies, such as X-rays or PET scans, to look for bone lesions or other signs of MM [2].

Treatment for MM typically involves a combination of chemotherapy, targeted therapies, and stem cell transplantation. The specific treatment plan will depend on the individual patient and the stage of their disease, as well as on molecular analysis that determines the risk to help control the growth of the cancerous plasma cells and alleviate symptoms [1]. In some cases, MM can be put into remission, but it is typically considered a chronic disease that requires ongoing treatment [4].

MM is a relatively uncommon cancer, accounting for about 1% of all cancers. It is common in people over the age of 65, and it is more common in men than in women [5]. According to estimates, there is a 1% annual risk of developing MM or a condition that is associated with it [5]. One of the most widespread pre-malignancies, smoldering myeloma, has a higher risk of transforming into MM. Monoclonal gammopathy of undetermined significance (MGUS) affects 3% of white people 50 years of age or older and nearly twice as many African Americans [6]. Several clinical and laboratory characteristics are commonly seen in patients with MM, including elevated levels of monoclonal immunoglobulins in the blood or urine, anemia, bone lesions, hypercalcemia, and kidney problems [4].

MM can cause anemia, which is an underproduction of red blood cells (RBC) due to excessive plasma cells in the bone marrow that can lead to symptoms such as fatigue, shortness of breath, and weakness. High levels of calcium in the blood owing to MM can lead to a condition known as hypercalcemia that can potentially cause thirst, fatigue, and confusion. Furthermore, MM can cause lytic bone lesions, or areas of abnormal bone growth, which can lead to bone pain and a higher risk of fractures. MM can also cause kidney issues and neurological symptoms, including weakness or even numbness in some cases. It is noteworthy to note that not all patients with MM will experience all of these clinical and laboratory characteristics, while the specific symptoms and complications will depend on the individual patient and the stage of their disease [3].

As people age, their risk of developing multiple myeloma rises. A high dietary intake of green vegetables and seafood has been linked to a lower risk of the disease, whereas obesity has consistently been linked to an increased risk of multiple myeloma [6]. The etiologic causes of white people having higher rates of multiple myeloma than African Americans have not been sufficiently researched [7]. The research identified that the African American population has a higher risk of developing MM than Caucasians. While these variables may be linked to either an increased or decreased risk of multiple myeloma, it is crucial to remember that other variables can influence a person's risk of getting the condition. Exposure to specific chemicals, radiation exposure, and specific inherited genetic abnormalities are other possible risk factors for multiple myeloma [7].

MM is not considered to be a genetic disease, but research has shown that there is a slightly higher risk of developing MM in individuals with a family history of the disease [2]. This increased risk is thought to be due to a combination of genetic and environmental factors. Smoldering myeloma is a precancerous condition that alters certain proteins in the blood and/or increases plasma cells in the bone marrow, but it does not cause symptoms of the disease. MGUS is a condition in which a person has an abnormal protein called a monoclonal protein in their blood [3]. It is considered to be a precursor to MM and is often asymptomatic. People with MGUS have a slightly increased risk of developing MM, as well as other types of cancer, such as Waldenström macroglobulinemia and chronic lymphocytic leukemia [3]. It is important to note that the majority of cases of MM occur in individuals without a family history of the disease. Therefore, having a family member with MM does not necessarily mean that a person will develop the disease [1]. However, it may be advisable for individuals with a family history of MM to discuss their risk with a healthcare provider and consider undergoing regular screenings for early detection [5].

The survival rate for multiple myeloma varies depending on various factors, including the stage of the disease at diagnosis, the patient's age and overall health, and the treatment options available. According to the American Cancer Society, the five-year survival rate for multiple myeloma is about 50%, meaning about half of all people diagnosed with multiple myeloma are still alive five years after their diagnosis [8]. However, it is important to note that survival rates are estimates and can vary widely depending on the individual circumstances of each case. The prognosis for multiple myeloma has improved significantly in recent years due to advances in treatment options [9]. Newer treatments, such as targeted therapies and immunotherapies, have shown promising results in improving survival rates and quality of life for people with multiple myeloma. Individuals with multiple myeloma need to work closely with their healthcare team to determine the most appropriate treatment plan for their specific situation [10].

This study aims to assess the clinical and laboratory characteristics of patients suffering from MM in King Abdulaziz Medical City-National Guard.

## Materials and methods

This is an observational study of multiple myeloma patients who were admitted to King Abdulaziz Medical City - National Guard, from January 2015 to December 2020. Patient records were reviewed to derive clinical and laboratory characteristics. Descriptive data analysis and survival analysis were obtained using SPSS (IBM Corp., Armonk, NY).

Study design, area, and settings

This is an observational retrospective cross-sectional study of patients admitted to King Abdulaziz Medical City - National Guard Governmental Hospital in Riyadh, specifically at the hematology and oncology department. KAMC-R, a tertiary care center, is one of the most distinguished hospitals in Saudi Arabia that receives consultations nationwide. The study underwent ethical approval from the ethical review committee of King Saud bin Abdulaziz University for Health Sciences. The Institutional Review Board (IRB) approved the study with the number SP20/263/R. This approval allowed us to proceed with research in compliance with ethical guidelines and ensured the protection of participants' rights and welfare.

Identification of study participants

This study included all multiple myeloma patients who were admitted to King Abdulaziz Medical City in Riyadh over a half-decade period from January 2015 to December 2020. The International Myeloma Working Group (IMWG) criteria were used to diagnose the patients. Both males and females of all group ages and nationalities who were diagnosed with multiple myeloma from 2015 to 2020 were included in this study. Those with plasma cell reactions to connective tissue disorder, liver disease, carcinoma, and chronic infection were excluded from the study. A simple random sampling technique was applied to select the diseased patients [9].

Based on our exclusion criteria, patients who were diagnosed with plasma cell reactions to connective tissue disorders, liver diseases, carcinomas, and chronic infections were excluded because they were not the target of this study.

This study aims to obtain an estimation of the prevalence of certain characteristics in multiple myeloma patients. Worldwide, the five-year limited-durability prevalence of multiple myeloma is nearly 230,000 patients [11]. In the United States, an estimated 30,330 people were newly diagnosed with multiple myeloma, resulting in 12,650 deaths [5]. Annually, about 20 to 30 patients with multiple myeloma get admitted to King Abdulaziz Medical City in Riyadh. With a marginal error of 5%, a confidence level of 95%, and a response distribution of 50%, the calculated sample size was 152.

Data collection process

The data were collected from King Abdulaziz Medical City (KAMC) files of patients diagnosed with multiple myeloma. The main predictors were abstracted from patients’ files using a data collection sheet that includes all the patients’ demographics like age, gender, nationality, and laboratory findings. The values of laboratory tests were obtained from the tests performed when the patient first presented to the hematology/oncology department. Moreover, clinical data such as date of diagnosis, presenting symptoms, and treatment were used to derive the outcome variable as an estimation of the prevalence of the mentioned predictors among MM patients. Data regarding cytogenic abnormalities (FISH data) were not included. Patient confidentiality was conserved by replacing the MRI number with a serial number. Only investigators have access to the secure files where patients’ data is kept.

Data were scored as high, normal, and low. Dates of birth, diagnosis, and death/last seen were used in statistical analysis. The normal range established for all variables mentioned in Table 1 served as the basis for data points. Within the normal range, values were considered normal. Any value below the normal range was considered low, while any value above the normal range was considered high. These definitions ensured that we accurately assessed and categorized the data.

**Table 1:** Normal range of variables

Variables	Normal range
Ca	2.1–2.55 mmol/L
BUN	3–9.2 mmol/L
Hgb	135–180 g/L
Hct	0.42–0.54 L/L
Wbc	4.00–11.00 × 10^9^/L
Plt	150–400 × 10^9^/L
IgG	7.51–15.60 g/L
IgA	0.82–4.53
IgM	0.46–3.04 g/L
Free kappa	19.40 mg/L
Free Lambda	5.71–26.30 mg/L
Free kappa/Lambda ratio	0.26–1.65

Data analysis

Our data have been entered by the co-investigators using Microsoft Excel (Microsoft® Corp., Redmond, WA) and analyzed using SPSS (IBM Corp., Armonk, NY) [12]. We presented categorical variables as frequencies and percentages, and numerical variables as mean ± standard deviation. We used the Pearson Chi-square test to assess the relationship between MM characteristics and the various predictors. The Kaplan-Meier test was used to estimate the survival time for MM patients in years. Crude and adjusted hazard ratios were computed to identify the factors that are related to survival while adjusting for other relevant covariates. Univariate analysis was done to show the distribution of the different variables, which include gender, age, and survival. Multivariate analysis was done by computing crude and adjusted hazard ratios using the Cox proportional hazard model. All P-values were two-tailed and a p-value of <0.05 is considered to be statistically significant.

## Results

A total of 151 patients were diagnosed with MM in NGHA from 2015 to 2020, with 95 (62.9%) being males and 56 (37.1%) being females. In Table 2, we categorized our patients into four groups according to their age, with the youngest ≤ 50 accounting for 14.6%, 21.9% of the patients between 51 and 60, almost 29.1% for those who were 61-70 years old, and the peak range was 71 years old or older, with a percentage of 34.4%. The mean age of diagnosis was determined to be 62.6 with a standard deviation of 13.4 (range = 32 to 95 years of age). The majority of our sample was from Saudi Arabia (90.2%), while the rest (9.8%) were from other countries. Death rates throughout the time of study conduction were 19.2%, accounting for a total of 29 patients, and the median overall survival from the time of myeloma diagnosis was 5.1 years with a 95% confidence level.

**Table 2:** Characteristics of patients having multiple myeloma

Variable		Frequency (percentage)
Gender	Female	56 (37.1%)
	Male	95 (62.9%)
Age	≤50	22 (14.6%)
	51–60	33 (21.9%)
	61–70	44 (29.1%)
	71+	52 (34.4%)
Survival	Alive	122 (80.8%)
	Dead	29 (19.2%)

**Figure 1:**
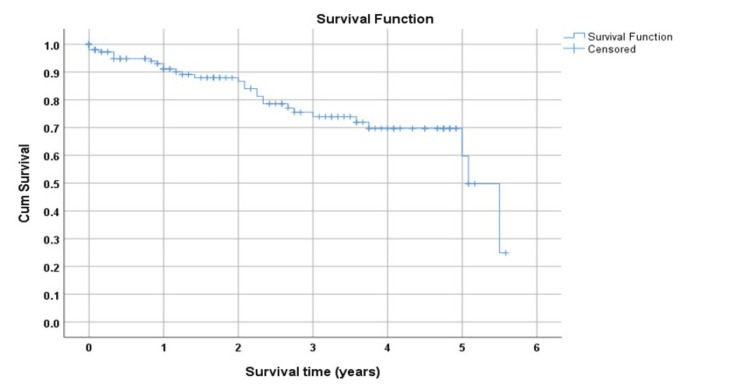
The Kaplan Meier curve for the multiple myeloma patients

The median overall survival from the time of myeloma diagnosis was 5.1 years, with a 95% confidence level. The median follow-up period is every three months. To determine the median overall survival time using the Kaplan-Meier test, we need specific data on survival times for individuals in a study or population. Without the actual data, it is not possible to provide an accurate median overall survival time. The Kaplan-Meier test is a statistical method used to estimate survival probabilities over time, but the specific median value can only be calculated with the actual survival time data. The survival curve for these patients is shown in Figure 1.

The characteristics of laboratory results are listed in Table 3. Regarding lab results, none of the patients had high levels of hemoglobin, while 83% showed up to have low levels. For WBCs and platelet counts, most of the results were normal. However, 49.6% of the patients’ calcium levels were normal, 28.5% were low, and 22% were high. Concerning the immunoglobulin and light chains, subtypes of myeloma, the most common type was IgG, 106 out of 147 (46.5%), the kappa chain, 96 out of 147 (65.8%), and the lambda chain, 46 out of 147 (31.5%).

**Table 3:** Characteristics of multiple myeloma patients by survival status

Variable	N	Alive, N = 122 frequency (percentage)	Dead, N = 29 frequency (percentage)	p-value
Gender	151			0.025
Female		40 (32.8%)	16 (55.2%)	
Male		82 (67.2%)	13 (44.8%)	
Age	151			0.3
≤50		16 (13.1%)	6 (20.7%)	
51–60		29 (23.8%)	4 (13.8%)	
61–70		38 (31.1%)	6 (20.7%)	
71+		39 (32.0%)	13 (44.8%)	
Nationality	132			0.5
Non-Saudi		12 (11.1%)	1 (4.2%)	
Saudi		96 (88.9%)	23 (95.8%)	
Hb	143			0.6
Low		96 (82.8%)	24 (88.9%)	
Normal		20 (17.2%)	3 (11.1%)	
WBC count	141			>0.9
High		14 (12.3%)	3 (11.1%)	
Low		32 (28.1%)	9 (33.3%)	
Normal		68 (59.6%)	15 (55.6%)	
Platelets count	139			0.9
High		9 (8.0%)	1 (3.8%)	
Low		32 (28.3%)	7 (26.9%)	
Normal		72 (63.7%)	18 (69.2%)	
Calcium levels	122			>0.9
High		22 (22.7%)	5 (20.0%)	
Low		27 (27.8%)	8 (32.0%)	
Normal		48 (49.5%)	12 (48.0%)	
Chemotherapy	148	93 (78.2%)	21 (72.4%)	0.5
Radiotherapy	151	11 (9.0%)	1 (3.4%)	0.5
BMT surgery	150	50 (41.3%)	11 (37.9%)	0.7
Anemia	150	40 (33.1%)	17 (58.6%)	0.011
Bortezomib	144	21 (17.8%)	5 (19.2%)	0.8

**Figure 2:**
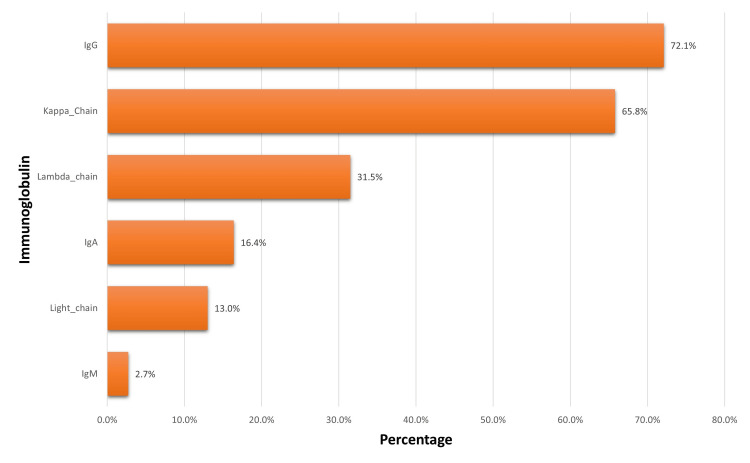
The laboratory findings of our patient’s immunoglobulins type

**Figure 3:**
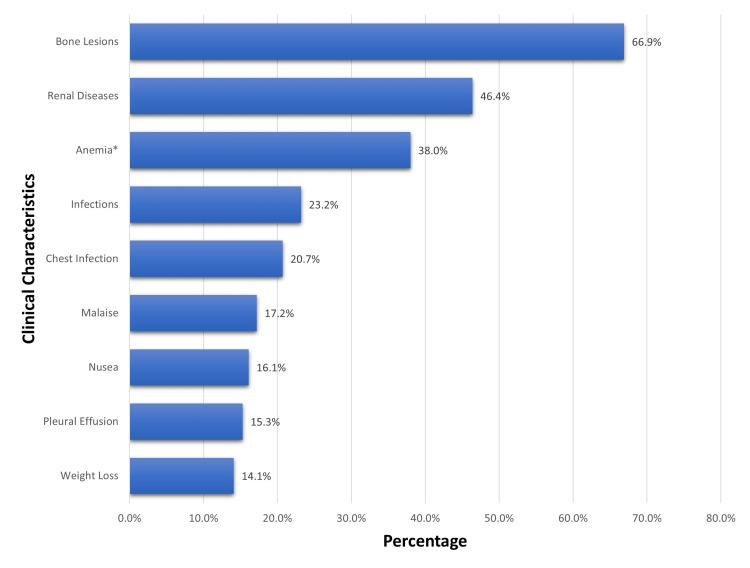
The clinical characteristics of the participants included in this study

The most common immunoglobulins were IgG, found in 72.1% of individuals and Kappa chain, found in 65.8% of the patients (Figure 2). The clinical characteristics are shown in Figure 3. Among 151 MM patients, the most common signs were bone lesions and renal disease, with a percentage of 66.90% and 46.40%, respectively. The third most common manifestation was anemia, which was present in 38% of patients. Almost half of the patients had comorbidities, as the prevalence of diabetes was 50.3% and hypertension was 48.3%. The treatment of choice for most patients was chemotherapy (77%), followed by autologous bone marrow transplant (40.7%). There was no allogeneic transplant. The majority of the patients had one autologous transplant and three patients had a double transplant. Only 12 out of 151 (7.9%) patients had radiotherapy treatment. Among the patients who underwent chemotherapy, the most common regimens were VCd (39.3%) and VRd (35%). Males had a lower hazard ratio as compared to females but this difference disappeared in the adjusted analysis. Patients with anemia had a significantly higher hazard ratio in both unadjusted and adjusted analyses (aHR = 2.61; 95% CI = 1.21-5.65) (Table 4).

**Table 4:** Crude and adjusted hazard ratios

Variable	Univariate		Multivariate	
	Crude HR	95% CI	Adjusted HR	95% CI
Gender				
Male	0.45	0.21–0.94	0.53	0.25–1.15
Female	1		1	
Anemia				
Yes	2.92	1.36–6.27	2.61	1.21–5.65
No	1		1	

## Discussion

Our study revealed that the disease manifests itself at an average age of 62.6, with over one-third of patients being diagnosed after the age of 70 and more than half of the patients being men. To the best of our knowledge, there are not many papers describing the epidemiology of MM in Saudi Arabia, but research conducted elsewhere throughout the world has found patterns that are similar to Saudi Arabia's, including greater incidence rates among men than women and a median age at diagnosis of 70 years [7,10,13].

Similar to the results of prior investigations, immunoglobulin G and the kappa light chain were the most prevalent among our individuals, followed by IgA [6]. IgM, on the other hand, made up a relatively minor portion (2.7%). No correlation between immunoglobulin subtypes and age of onset was found using Pearson chi-square testing. Despite finding normal to low serum calcium levels, which are comparable to results from an Egyptian study, several investigations reported hypercalcemia as a key finding [10]. Compared to the Mayo Clinic trial, there was evidence of a higher percentage of thrombocytopenia [14].

In line with prior research, symptomatic patients reported bone marrow lesions most frequently at the hip, vertebrae, and other locations [15]. This may be explained by osteoblastic bone resorption, which is brought on by bone marrow plasma cells and may eventually result in pathological fracture, bone pain, and osteoporosis [16]. The majority of patients with multiple myeloma had hemoglobin levels below the threshold of 135 g/L, which was considered normal. This might be brought on by CKD, bone marrow suppression, nutritional problems, or aging. Many patients' only initial manifestation is a lab abnormality, such as anemia, renal dysfunction, or increased protein [17]. Nearly all individuals with multiple myeloma have anemia at some point in the course of the illness.

A population-based study conducted on 9253 MM patients revealed that MM patients have a 7 times higher risk of contracting infections compared to the control group. Chest infections, such as COVID-19 and Klebsiella, as well as other infections with Gram-negative bacteria as the predominant causal organism, e.g., E. coli, and salmonella, were as common as 23.20% of the MM patients in our study. Although it is frequently associated with MM [17], weight loss was the clinical symptom that was least frequently mentioned. In individuals with MM, hypertension and type 2 diabetes are frequently documented. These conditions may be brought on by advanced age, complications from the disease, or side effects from MM medications [18].

Except for the association between survival rates and gender, the clinical and laboratory features in the survival study were not statistically significant [19]. After the trial, 16 out of 56 female patients and 13 out of 95 male patients had died. Gender can therefore be thought of as an unintentional predictive factor for MM patients' overall survival.

A risk factor for multiple myeloma has been discovered as a family history of cancer, particularly lymphohematopoietic cancers, which account for nearly 15% of this disease [10]. The specific pathways, however, through which a family history of cancer can raise the risk of multiple myeloma are unclear and may result from a confluence of genetic and environmental variables [16]. The likelihood of developing multiple myeloma has been linked in certain studies to specific genetic variants; however, more study is required to validate these results and to pinpoint the precise role that genetics plays in the disease's onset [20]. It is crucial to understand that while a family history of cancer or specific genetic variants may raise a person's chance of developing multiple myeloma, this does not guarantee that they will get the illness [21]. The chance of having multiple myeloma can also be influenced by numerous additional elements, both genetic and environmental [22]. Given that there are currently no reliable, consistent, or established predictors of the risk of developing multiple myeloma, more efforts should be directed toward identifying the cause in addition to understanding the characteristics or symptoms of the disease [23].

One of the limitations of our study was its study design, as retrospective studies are typically useful in identifying trends or associations between several factors, but they are not always as reliable as prospective studies [24]. The findings can be limited by the quality and completeness of the available data, as well as the potential for bias or confounding factors that may have influenced the results [25]. In the case of a small sample size, the results of the study may not be representative of the larger population and may not be generalizable to other groups of people [26]. Additionally, attrition is also typically high in retrospective studies [27]. For instance, 16 female patients out of 56 passed away at the end of the study duration, compared to only 13 male patients out of 95. Therefore, gender can be considered an incidental prognostic factor for the overall survival of MM patients in our study.

Retrospective studies are frequently helpful in finding trends or associations between various characteristics, but they are not necessarily as accurate as prospective studies, which was one of the limitations of our study [28]. The accuracy and comprehensiveness of the available data, as well as the possibility of bias or confounding variables that may have influenced the results, can all restrict the conclusions. The study's findings might not be indicative of the larger community in cases where the sample size is limited, and they might not be transferable to other populations [29]. Furthermore, in retrospective research, attrition is frequently high. Retrospective studies also frequently experience substantial attrition [15]. Similarly, 16 out of 56 female patients died at the end of the research, compared to just 13 out of 95 male patients. Therefore, in the context of our investigation, gender can be regarded as an accidental predictive factor for MM patients' overall survival.

## Conclusions

A thorough history focused on symptoms should be taken whenever any of these results or symptoms are reported to diagnose and treat the disease as quickly as possible. Clinical presentations like bone lesions, renal diseases, and anemia are often the only initial presentations in many patients. This study comprised individuals diagnosed with multiple myeloma for six years, with a sample size of 120. We recommend future research into multiple myeloma be done over a longer period of time with a larger sample size for a clearer understanding of the condition.
