# Correction: Descriptive Characteristics of Multiple Myeloma Patients in King Abdulaziz Medical City National Guard

**DOI:** 10.7759/cureus.c159

**Published:** 2024-02-05

**Authors:** Sultan Alqahtani, Lama Alyabis, Hissah Alyabis, Nouf Al Qurashi, Rose Almadi, Majd Alsoman, Mohsen Alzahrani

**Affiliations:** 1 Department of Basic Medical Sciences, King Saud Bin Abdulaziz University for Health Sciences, College of Medicine, Riyadh, SAU; 2 Research, King Abdullah International Medical Research Center (KAIMRC), Riyadh, SAU; 3 College of Medicine, King Saud Bin Abdulaziz University for Health Sciences, Riyadh, SAU; 4 Department of Oncology, Division of Stem Cell Transplantation and Cellular Therapy, King Abdulaziz Medical City, Riyadh, SAU

This article has been corrected by the journal as it was originally published with the wrong discussion section due to a technical error made during the final editing process. The journal deeply regrets this error.

